# Forging hospital and community partnerships to enable care coordination for opioid use disorder

**DOI:** 10.1186/s13722-025-00565-y

**Published:** 2025-04-24

**Authors:** Zoe Lindenfeld, Berkeley Franz, Alden Yuanhong Lai, José A. Pagán, Cheyenne Fenstemaker, Cory E. Cronin, Ji Eun Chang

**Affiliations:** 1 Edward J. Bloustein School of Planning and Public Policy, Rutgers University, New Brunswick, NJ 08901 USA; 2 Heritage College of Osteopathic Medicine, Ohio University, 1 Ohio University, Athens, OH 45701 USA; 3 Department of Public Health Policy and Management, School of Global Public Health, New York University, 708 Broadway, New York, NY 10003 USA; 4 College of Health Sciences and Professions, Ohio University, 1 Ohio University, Athens, OH 45701 USA

**Keywords:** Opioid use disorders, Substance use disorders, Hospitals, Care coordination, Partnerships, Care transitions

## Abstract

**Background:**

Programs that aim to increase access to substance use disorder (SUD) treatment in hospital-based settings have proliferated in recent years. These efforts include transitional opioid programs (TOPs), which navigate patients to community-based SUD treatment programs post-discharge. Successful navigation from TOPs to outpatient treatment hinges on effective coordination between hospitals and post-discharge endpoints, yet it is unclear how hospitals can best develop effective partnerships with outpatient treatment organizations. The objective of this study is to synthesize the common themes underpinning the development of partnerships to facilitate care transitions between TOPs and ongoing SUD treatment.

**Methods:**

Qualitative study with staff and providers from hospitals affiliated with four safety-net health systems (*n* = 21) and leaders from community-based organizations (CBOs) and treatment facilities that had established referral partnerships with one of the four health systems in our study (*n* = 4).

**Results:**

Analysis of interview transcripts revealed seven common themes that underpinned the development of care transitions partnerships: (1) Active, intentional outreach; (2) Responding to a community need; (3) External Enabling Factors; (4) Leveraging reputations and community connections; (5) Focusing on operations; (6) Reciprocal relationships; and (7) Building Infrastructure and Processes to Ensure Collaboration. The seven identified themes were categorized into three groups corresponding to different partnership development stages. The first group revolves around the initial stage of meeting and developing a relationship (themes #1–4). The second set focuses on navigating and resolving challenges that arise in the partnership (themes #5–6). Lastly, the third group pertains to sustaining a partnership long-term (theme #7).

**Conclusions:**

This study identifies seven core themes underlying the development of care transition partnerships for SUD patients within four safety net health systems and their CBO partners. These themes demonstrate how partner organizations can establish the trust, reciprocity, and commitment necessary to support patients through the critical transition period.

**Supplementary Information:**

The online version contains supplementary material available at 10.1186/s13722-025-00565-y.

## Background

Although research indicates that evidence-based treatment improves outcomes for individuals with opioid use disorders (OUDs), rates of treatment receipt remain low. In 2021, despite multiple federal and state efforts to improve access to effective, evidence-based medication for opioid use disorder (MOUD) treatment [1, 2], less than 10% of individuals with a substance use disorder (SUD) received any treatment [3]. Given that deaths due to drug overdose continue to rise, with over 107,000 deaths recorded in 2022 [4], interventions that enhance treatment access amongst individuals with SUDs are critical.

Hospitals are a key entry point into the healthcare system for patients with SUDs [5]. Protocols for emergency medical services encourage patients suspected of a drug overdose to be transported to the emergency department (ED) for evaluation [6, 7], and there has been a 99.4% increase in overdose-related ED visits over the past decade [8]. Within inpatient settings, approximately one in five patients on a general medicine ward have an SUD [9], many of whom remain disengaged from routine primary care or ongoing SUD treatment [10, 11]. Overall, it is estimated that SUDs cost U.S hospitals $95 billion annually, accounting for 8% of all hospital expenditures [12]. In response, programs that aim to increase access to SUD treatment in ED and hospital settings have proliferated in recent years [13, 14]. Examples of these programs include addiction consult teams, which initiate MOUD treatment such as buprenorphine in the ED or inpatient setting; [9, 15] ED-based bridge clinics, which provide rapid MOUD initiation, stabilization, harm reduction services, and linkage to outpatient providers; [16] peer programs, which provider recovery support and mentoring to patients with SUD in both the ED and hospital settings; [17] and transitional opioid programs (TOPS), which navigate patients to community-based SUD treatment programs post-discharge [9, 15]. 

A key factor in the success of both addiction consult teams and TOPs is patient linkage to ongoing treatment in community-based settings following discharge [18]. Given that discharge is a particularly vulnerable time for patients with SUDs, with hospitalized SUD patients having higher rates of patient-initiated discharge [9, 19], improving care transitions for patients with SUD is a critical yet difficult task [18]. Prior studies focusing on evaluating TOPs in hospitals emphasize the necessity of staff roles dedicated to SUD care coordination, such as care managers or peer specialists [10, 20], psychosocial support [18], as well as the importance of addressing the non-medical needs of patients (e.g., lack of transportation access, unstable housing) that impede patients from completing referrals to outpatient treatment [18, 21]. 

Successful navigation from TOPs to outpatient treatment hinges on effective coordination and communication between hospitals and post-discharge endpoints [18, 22], yet it is unclear how hospitals can best develop effective partnerships with outpatient treatment organizations. Previous research on clinical-community partnerships highlights the importance of trust, rapport, and timely communication in forging these relationships [23]. Research on Accountable Care Organizations (ACOs), which are groups of providers that voluntarily partner to provide coordinated care to largely vulnerable patient populations [24], has discussed the utility of data sharing [25, 26], resource complementarity [27], formal or informal agreements [27], and existing social ties [25, 28], in developing new ACO partnerships. In the absence of a pre-existing relationship, studies have also highlighted the importance of transparency among ACO partners, as well as the benefits of having a neutral facilitator support the development of the relationship [27]. However, no prior study has specifically evaluated the key elements underpinning the development of partnerships between hospitals and community-based organizations (CBOs) across the SUD treatment care continuum.

In evaluating the critical components of hospital-CBO partnerships, it is especially important to focus on safety-net hospitals, which disproportionately serve ethnic/racial minority and uninsured patients and have high rates of SUD-related admissions [13]. Because safety-net hospitals operate on limited budgets that restrict the availability of resources required to sustain successful implementation efforts, such as funding and staffing initiatives, these settings face amplified challenges to implementing TOPs [29, 30]. These challenges may, in turn, lead to collaborative patterns that are distinct from those of hospitals facing fewer resource constraints. Yet, most research on TOPs has focused on academic hospital settings [18]. As such, understanding how safety-net hospitals forge partnerships that transition patients with SUD to ongoing treatment post-discharge is essential to support the delivery of hospital-based TOPs in safety-net settings and to ensure equity in access to SUD treatment.

This qualitative study uses interviews with healthcare professionals, hospital leaders and staff, and directors of CBOs to synthesize the common themes supporting the development of partnerships to facilitate care transitions between TOPs in safety-net hospitals and ongoing SUD treatment. Given the barriers individuals with SUDs face in accessing treatment post-hospital discharge, it is important to understand how these settings can establish relationships that support the successful transition of SUD patients into community-based treatment [31]. These findings will provide essential insight for improving care coordination and treatment receipt rates for patients with SUD.

## _Methods_

### Study settings and participants

Data were collected as part of a larger study on the implementation of transitional opioid programs among safety net hospitals in the U.S. Study participants included staff and providers from hospitals affiliated with four safety-net health systems: (1) City Health (*n* = 4) based in Chicago, Illinois; (2) Lake Health (*n* = 6) in Central Ohio; (3) Metro Health (*n* = 6) based in New York City; (4) Mountain Health (*n* = 5) based in Southeastern Ohio. Health systems were given pseudonyms to protect the identity of interview participants. A description of the four study sites and their TOP referral endpoints can be found in Table 1. Participants also included leaders from community-based substance use treatment facilities and other CBOs that had established referral partnerships with one of the four health systems in our study (*n* = 4). Health systems were selected based on organizational and community characteristics to ensure diversity in the type of safety net hospitals included in the study. Leaders of CBOs were referred to the study team by their hospital partners. Participants from both the health systems and CBOs were recruited based on discussions with leadership from each site. Participants were eligible for inclusion if they were involved in treating, administering, or coordinating care for individuals with SUD in the hospital or CBO setting. Each health system provided consent for participation in the study in addition to the individual interviewees. Participants represented the spectrum of roles involved in facilitating clinical partnerships, and included staff in administrative, clinical, and non-clinical support roles.

**Table 1:** Description of participant health system sites and TOP referral endpoints noted by interviewees

Health System	Description	TOP Referral Endpoints
City Health	A network of safety net hospitals, community health clinics, and public health departments serving Chicago, Illinois. They have served their community for nearly 200 years and continue to grow and expand services to meet critical community needs such as opioid use disorder.	- Community based providers - Residential treatment programs - Intensive outpatient programs (IOPs) - Detoxification services - Community harm reduction programs - Outpatient programs (including MOUD)
Lake Health	Includes one safety net hospital located in central Ohio and a network of outpatient clinics. They are the largest healthcare provider in a six-county region which is considered medically underserved, and as such, they provide essential health services. They serve predominantly rural populations, heavily impacted by opioid use disorder and overdose.	- Outpatient programs (including MOUD) - Residential treatment programs - Behavioral health providers - Counseling programs - Inpatient facility for psychiatric and substance use patients - Detoxification services - Local mental health center
Metro Health	The largest municipal health care delivery system in the country. This health system includes 11 acute care hospitals as well as Gotham Health, a Federally Qualified Health Center which is the largest in the country and has clinics located across NYC. NYC Health + Hospitals provides health care services to more than one million adults and children every year.	- Outpatient methadone clinics - Outpatient providers (including MOUD) - Faith-based treatment programs - Residential treatment programs - Detoxification services - Community harm reduction programs
Mountain	A safety net hospital located in the Appalachian region of Southeastern Ohio. As a member of the Mountain Health system, this hospital organization provides acute- care services and outpatient medical care to the surrounding community. Opioid use disorder has affected the surrounding counties greatly; nearby River county has the highest opioid overdose rates in Ohio and is among the highest in the U.S.	- Outpatient providers (including MOUD) - Residential treatment programs - Detoxification services - Outpatient methadone clinics - Counseling services

### Data collection and measures

Data collection began in November 2022 and continued through August 2023. We gathered data through semi-structured interviews. We developed an initial interview guide with 15 questions divided into five categories: general background, opioid use in the community, TOPs, facilitators to implementing TOPs, and barriers to implementing TOPs. We also used interview probes that focused on the history and motivation behind established partnerships, short-term vs. longer-term engagement with partners, and how organizations maintained partnerships. The full interview guide can be found in Appendix Table 1. Following common procedures for semi-structured studies [32], we utilized follow-up questions to explore participants’ statements in detail, tailoring our approach to their unique expertise and role. Interviews continued until we reached theoretical saturation, meaning that no new insights or themes emerged from the interviews.

All interviews were conducted by at least two study team members, with one leading and the second taking notes. Interviews were 45–60 min in length and conducted via Zoom, and were recorded, professionally transcribed, and de-identified. Following interviews with health system participants, we followed up over email to ask for CBO partners’ names and contact information for the study team to interview. All participants were compensated with $50 gift cards, and approval for this study was obtained from the _____University Institutional Review Board.

### Data analysis

Data analysis began with two rounds of coding with progressive refinement. First, all study team members coded a transcript to create a codebook. All study team members created their own codes as they coded, though the structure of the interview guide guided the initial coding exercise. We discussed similarities and differences, and generated codes with accompanying descriptions of how they should be used in the coding process. Following this step, two study team members (ZL and CF) coded each interview transcript. We then used an inductive, modified Grounded Theory-based [33] approach to qualitative data analysis. To identify interview texts focused on developing partnerships, as well as factors that enable partnership development, ZL extracted segments of interview texts that had been open-coded with the codes ‘Facilitator’ and ‘Partnership’ during the initial rounds of multi-person coding, and recoded these segments using more detailed and meaningful codes. ZL then grouped these codes thematically, asking “whether the emerging themes suggest concepts that may help us describe and explain the phenomena we are observing” [34]. Analysis was conducted at the organizational-level, and themes were identified by examining patterns across responses from hospitals and CBOs. Upon identifying the 7 themes, we recognized their temporal connections, so we set out to analyze and categorize them into phases. A data structure that demonstrates how the main themes uncovered in our study emerged from underlying codes can be found in Table 2.

**Table 2:** Data structure showing categories derived during open coding and their associated themes

Theme	Underlying Code
**Active**,** intentional outreach**	Identifying the right stakeholder
	Being intentional with check-ins
	Someone dedicated to make these linkages
	Identifying a rolodex of partners
	Showing up in person to make connections
	Dedicated staff to forging and maintaining relationships
	Physical presence
	Actively building relationships with outpatient organizations
	Creating one-on-one relationships
**Responding to a community need**	Passion about addressing SUD in the community
	Addressing shared community needs
	Identifying community issues rather than individual issues as priorities
**External Enabling Factors**	External coordinator for liaisons
	Technology to facilitate linkages
**Leveraging reputations and connections to community**	Hiring based off their recommendations to have a connected system
	Lead by champion with community connections
	Outcomes bring trust
	Community roots and network
	Reputations facilitate connections
	Leveraging long term relationships
	Reputation and referral networks
**Focus on operations**	Creating business relationships
	Operational conversations
**Reciprocal Relationships**	Identifying how we can help them
	Responsiveness is a two way street
	Helping each other
	Collaborative Approach, not just making demands
	Understanding what each partner needs from you
	Ensuring we have what the organization needs
	Relationships built on trust
	Open door policy on referrals
**Sharing resources and infrastructure**	Shared data systems
	Data sharing
	Co-locating employees

## Results

**Fig. 1:**
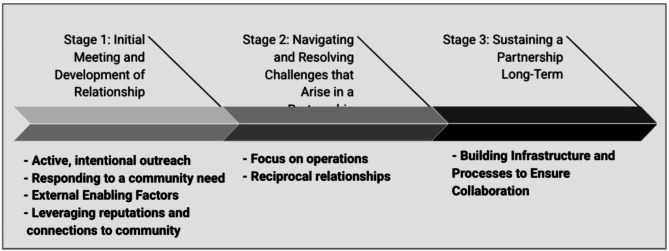
Themes underpinning the development of transition partnerships by relationship stage

Our analysis revealed that partnerships to transition patients from TOPs to ongoing SUD treatment in CBOs were perceived positively rather than as a potential source of conflict, and seven common themes underpinned the development of such partnerships: (1) Active, intentional outreach; (2) Responding to a community need; (3) External Enabling Factors; (4) Leveraging reputations and community connections; (5) Focusing on operations; (6) Reciprocal relationships; (7) Sharing resources and infrastructure. These seven themes were categorized into three groups corresponding to different partnership stages. The first group revolves around the initial stage of meeting and developing a relationship (themes #1–4). The second set focuses on navigating and resolving challenges that arise in the partnership (themes #5–6). Lastly, the third group pertains to sustaining a partnership long-term (theme #7). See Fig. 1 for the seven themes organized by stage of relationship development and Table 3 for additional quotations from our interviews organized by theme.

**Table 3:** Additional quotations from interviews organized by theme

Theme	Quote
Active, intentional outreach	“You can know who the director is. But if you want to get Mary or Johnny into the program, it’s good to know who the impact person is to expedite the process rather than just looking at a name on a directory, which would be my name, but I may not be the best person to call. You might need the person down the hall. So it’s about maintaining those relationships that makes the hand-off more friendly and quicker.” (Metro Health ID 1, clinical role) “When I first started building my relationships, I would just call around. I would literally go to relink.org and I would call agencies just at random and question and talk to them about what their services were, what patients they were willing to accept, age group, payer sources. That’s how I built most of my relationships was just randomly calling agencies here and there and talking over the phone with them.” (Lake Health ID 2, clinical role) “I really did some cold calling six years ago when I started… So when I got in the position, it was really, okay. So who does residential? Who does detox? Who does IOP? Who does OP? And calling, hi, my name is [name]. I’m calling from Cook County. Tell me a little bit about your program. And then it’s like, okay, well I’m going to be seeing patients in the emergency room and I would love to connect them to your organization. Give me a point person, what’s their extension? What do you need me to fax you? I have a release of information. What else do we need to do? And it really, six years later, and I’m still in communication with most of those treatment facilities, now you have the resolving door people that leave those organizations turnover. But as far as the partnership and making sure that the patients get into a higher level of care, that’s still there. But it started from really cold calling. I don’t know. I was like, I’m just going to go down this Rolodex here and see what happens.” (City Health ID 3, non clinical staff role) “We go to these events, and the events that we go to, usually there are many, many tables of other facilities, that they get up and they say, “Okay, this is what we offer.” And [name] and I sit there in the background saying, “That would be a great place.” So we go to that table, we talk to those people, we get their cards, we get their information… So these events that we go to, I think, is our biggest source of information” (Mountain Health ID 1, clinical support role)
Responding to a community need	“The whole community was on board. They seen there’s a need, even though it’s several different facilities, we’re all in it for the same reason. And there are plenty of people with addiction to go around, unfortunately.” (Mountain Health ID 1, non clinical support) “I think communication’s key. And these I see as not only individual or organizational issues to develop strategies around, we see it as a community problem and a community need. And so I think convening regular times or meetings when we can, as a community, sit down and talk about the issues of mutual concerns for all of us, and then work to develop strategies to impact that together versus working in our silos.” (Lake Health ID 7, administrative role at CBO)
External Enabling Factors	“The [Title] Network is one that allows us to connect, so I get referrals at my clinics from private universities and patients being referred through that, which is really wonderful so I could go both directions… it functionally is a website. The website basically reserves a day for follow-up with the patient. The patient, before they leave the emergency department, they know, “Okay, Wednesday the 23rd, you’re going to follow-up at this clinic.” It doesn’t give them a time, but it gives them at least a day.” (Metro Health ID 3, administrative role) “And the big thing obviously was to assure linkage into the community. So outside of the doctor meetings, then we had leaders from our community resources, which is our community board. And then two of our community agencies, they also joined our group. And what we set up was if our ER doc would prescribe the Suboxone to guarantee linkage out within 24 hours. So [the community board] set up a process to do that” (Mountain Health ID 5, clinical role)
Leveraging reputations and connections to community	“Over time, it’s our ability to engage people in the next step and then show the hospital what we’re doing to build that reputation and that comfort level.” (City Health ID 5, administrative role at CBO) “My honest opinion is those key players involved with the collaborations are long-term folks…Matter of fact, some of them I worked with since I was 19. I mean very, very long-term and committed folks in these positions. So I feel like that’s what helps the collaboration to continue across the years.” (Lake Health ID 1, administrative role) “I think the biggest thing that I could share in that regard…is that our current CEO used to be on the board of one of these community substance use programs.” (Metro Health ID 1, clinical role)
Focus on operations	“And then also making sure that there’s operations connections to work through kinks, and communication when something’s not working. Something might be really bugging us that they do, that they might be like, “We don’t have to do it that way.” (City Health ID 2, clinical role) “We’ll invite [our partners] to our service line meeting we have once a month, we’ll invite them in to talk about any challenges and they’ll be part of those conversations, which like I said, five to seven years ago that communication wasn’t there.” (Lake Health ID 3, clinical role) “We have a business relationship in the sense that methadone programs hear from us all the time because we’re verifying doses for patients that are here. They hear from us all the time because we’re trying to coordinate transition from inpatient to outpatient for a lot of people. And so we operate on a business level that way.” (Metro Health ID 1, clinical role) “So when we’d have our meetings, what they would talk about is, so, “What if we signed somebody up, say at Integrated Services, and they start there but we’re finding out maybe Health Recovery would’ve been a better choice.” Everyone talked about, “Well, how would that work? Would you be okay to transfer?” And people’s like, “Yeah, it’s about the patient.“” (Mountain Health ID 1, non clinical staff role)
Reciprocal relationships	“We have a community treatment provider that again offers a lot of the higher levels of care that we don’t. And so a lot of times patients will come to us for the medication, but we’ll go to them for the IOP, the residential, the whatever.” (City Health ID 1, administrative role) “We have agreements, professional agreements signed, collaborative agreements with certain entities. But really even without those, we would still work very closely together. They have services that we need for our patients and we have services that they need for their patients. So that’s what really drives that connection, I think.” (Lake Health ID 1, clinical role) “So we’re doing a joint meeting with [partner organization’s] staff and our staff… Because we don’t currently have inpatient detox, so we have relationships with many programs and we cross-mingle and refer to them and they refer to us.” (Metro Health ID 2)
Building Infrastructure and Processes to Ensure Collaboration	“Your specific subject on hospitals, we’ve had longstanding relationships with hospitals in Illinois. We’re currently in nine hospitals, and we’re providing a variety of services in those nine hospitals.” (City Health ID 5, administrative role at CBO) “We are completely separate entities and we outreach and connect and have created this informal collaboration where they provide the support and the structure.” (Metro Health ID 1, clinical role) “We are trying to align intentionally the work that we do so that we could leverage resources, leverage human capital and build stronger pathways for and reduce duplication of efforts as we try to do this together.” (Lake Health ID 7, administrative role at CBO)

### Stage: initial meeting and development of a relationship

#### Active, intentional, outreach

A major component of developing relationships with external organizations was performing active outreach to potential partners in the community. Interviewees from health systems and CBOs discussed techniques such as cold-calling, visiting potential partner organizations in person, inviting staff to lunch, attending regional conferences for SUD treatment providers, and handing out business cards. Interviewees also noted that it was useful to have employees dedicated to forging these relationships and emphasized the necessity of building rapport to make transitions friendlier and more efficient. As described by a participant from a health system:

“We actively went after other agencies to find, to build a rapport with them, so that we could get our patients in, we got inpatient, outpatient, but we needed to get them in quickly. And now, because we built such a good rapport, we’ll call, “Hey this is [name] or this is [Y] from Mountain” And they’re like, “Oh yeah… I don’t think we have any beds. Well, let me check. We’ll call you.” And usually we can get people in within the day, the same day, or the next day. So that building that rapport with your community and the patients.” (Mountain ID 1, non-clinical staff role).

Although it was useful to begin relationships by cold-calling, participants noted the importance of intentionally finding the right people to facilitate the partnership. This included staff in leadership roles with the capacity to formalize a relationship and frontline staff to facilitate communication and the care transition itself, including any administrative requirements. A health system participant noted:

“In my experience, sometimes the challenge is just identifying who is the right stakeholder. Who is the exact right person in this department, from this organization to really partner with who can move things for you if they are able to share the same vision that you have about supporting the patient population?” (City Health ID 1, administrative role).

Finally, participants highlighted the importance of actively sustaining relationships with partner organizations. This included scheduling regular check-in meetings to discuss challenges or new opportunities for collaboration, being consistently responsive to partners, as well as intentionally forging relationships with staff in long-term roles at a partner organization.

#### Responding to a community need

Interviewees discussed how framing a collaboration as a response to community needs, rather than the needs of a particular organization, enabled the successful development of hospital-CBO partnerships. Under this framing, strategies to transition patients with OUD/SUD between care settings were developed to address the context of the community served. This enabled health systems and CBOs to work outside of their organization’s individual concerns and find alignment in their missions and impact on the community. One CBO interviewee described their partnership, saying:

“I think it is more collaborative and we’re aligning our missions and visions. They’re a little more in alignment maybe than they have been in the past because I think we recognize that we can’t do this alone, that it’s going to take all of us to make an impact. I appreciate that about our communities and our partnerships.” (Lake Health ID 7, administrative role).

In developing an aligned mission, several interviewees described the utility of having community-wide meetings for all local SUD stakeholders and care providers. In these meetings, organizations were able to discuss issues of mutual concern, highlight high-priority or emerging issues, and build new connections and collaborations with potential partners. One CBO participant also noted that these meetings, which fostered a “we’re all in it together” ethos, enabled organizations to overcome fears of competition that previously served as a barrier to collaboration.

#### External enabling factors

Interviewees indicated that it was sometimes useful to have an external facilitators or supports to help develop a relationship between organizations. This included having a state or regional public health agency representative coordinate initial introductions or serve as a mediator when disagreements arose between partners. As noted by a health system participant:

“There are these SOR grants from SAMHSA, I don’t know if you’re familiar with them, but it’s state opioid response grants, and they basically funnel through the conduit of [state organization]. And [state organization] used these SOR grants to create regional networks. And in these regional networks, you have to have a hospital system as one of the partners. And so we are in a regional network, and we’ve partnered with several different organizations for referral sources and for different things. So that has worked out in some really good ways. Basically [the state organization] manufactures these regional partnerships where there has to be a lot of coordination between the partners. So that’s been, I think, a good thing for building some of those partnerships up.” (Metro Health ID 6, clinical role).

Participants from one health system also described how technology can facilitate relationship building. This included referral systems embedded into the EMR, such as the NowPow/UniteUs platform [35], as well as external websites that scheduled follow-up visits for patients being discharged from the ED at CBOs in the website’s network. Because this website was funded through the state health department, it offered vouchers for patients to use ride-sharing services for the care transition, reducing the burden on the health systems and CBOs to coordinate transportation for patients without vehicle access.

#### Leveraging reputations and connections to community

All health systems and CBOs described the significance of having positive reputations and connections to the local community in forging transition partnerships. To leverage reputations, organizations described practices such as hiring employees who previously worked at potential partner organizations, those with shared personal or employment histories with staff at other sites, as well as individuals with deep roots in the community. As stated by a health system participant:

“I feel like that’s a big thing because some of the people that work at the facilities that I have called say, ‘Oh, yeah. I used to work with you. I didn’t know you worked there,’ so it builds those bonds. I don’t know how else to explain it, but you know what I mean? Okay. Now I can make it easy on all of us. You know me, I know you.” (Lake Health ID 4, clinical role).

Having a reputation as an organization for doing good work and not turning away patients for not having insurance or the resources to pay also helped establish partnerships. For example, many participants described these partnerships as stemming from word-of-mouth referrals. One CBO participant also noted that their organization used data to demonstrate their success working with patients to health system partners. Finally, participants described the necessity of referencing successful prior experiences working together to establish a partnership. As one health system participant succinctly stated:

“The more my patients actually showed up, I think they [community-based treatment partners] trusted me a little more.” (Lake Health ID 2, clinical role).

### Stage: navigating and resolving challenges that arise in a partnership

#### Focus on operations

Several participants noted that maintaining a focus on day-to-day operations and practical concerns, rather than philosophical approaches to treating SUDs, enabled the development of successful transition partnerships. While participants noted differences in policies surrounding SUD treatment at partner organizations, they discussed how maintaining a focus on the implications for the patient, rather than the idealistic differences underlying them, fostered better communication between partners. For example, one health system participant described how their organization’s more liberal approach to MOUD dosing was at odds with the policies at a CBO, and that a resolution was reached to allow the health system to continue titrating patients via telehealth. Another health system participant discussed initial misunderstandings with a CBO partner due to a grant which required costly fentanyl testing for patients transferred to the CBO; however, once the purpose of testing was clarified, the two organizations were able to find mutual ground. As stated by a CBO participant:

“I’m public health trained, collaboration is key. That’s also where I train and work. But I have to trust that we have expertise and that we also understood their challenges, and that we were going to hear them and meet them, and try to enable solutions instead of just making demands on them.” (City Health ID 2, clinical role).

#### Reciprocal relationships

All health systems and CBOs interviewed described the importance of having reciprocal relationships based in mutual support with their partner organizations. This entailed having conversations early in the relationship surrounding what services were offered internally to their organization, and gaps in service delivery that could be potentially filled by partner organizations. This enabled the relationships to develop as beneficial to both partners, which promoted trust and support between the organizations. For example, one of the health systems interviewed continued to provide MOUD treatment to patients following a transition to a community-based intensive outpatient program (IOP) that did not have an MOUD-prescribing provider on site. Interviewees also discussed how these partnerships helped relieve stress amongst staff from both organizations, with one organization increasing support when the other was overburdened or inundated with patients. As described by an interviewee from a health system:

“It’s being able to really identify what resources our health system has, whether it’s data or clinical, and then what do our partners need and how can we support each other.” (City Health ID 1, administrative role).

Interviewees also noted the significance of data in developing reciprocal relationships, and demonstrating a gap in services or outcomes that could be supported by an external organization. One CBO used data on hospital readmission rates for SUD patients to advocate for the organization’s social workers to be embedded in the hospital’s ED. Another CBO used state health department data on overdoses to demonstrate the burden of substance use within the geographic area served by a hospital, and to make the case for a care transitions partnership. As noted by a CBO interviewee:

“We bring that data with us…And so when the hospital first says, well, we’ve got social workers, we’re taking care of this. Well, they’re really not okay. And we can really provide some data to show that some of these individuals are coming back several times, and the data is available.” (City Health ID 5, administrative role).

### Stage: sustaining a partnership Long-Term

#### Building infrastructure and processes to ensure collaboration

Participants noted how the ability to share data and other resources helped sustain successful care transition partnerships between CBOs and health systems. Participants from health systems described pursuing data sharing agreements with care coordinators from CBOs, sharing electronic medical record (EMR) systems with local jails (to facilitate linkage to care following a jail discharge), as well as developing formal referral systems that tie into the EMRs of health systems and CBOs. One health system also discussed how sharing staff with CBOs in their community enabled warm handoffs to occur, as well as the development of stronger relationships between organizations. As discussed by a health system participant:

“And where the relationship started, we had employees here, some even in the ED that worked at some of these facilities part-time, and that’s really where the relationships sprung from, employees that work at both sites.” (Lake Health ID 3, clinical role).

Aside from relationship building, participants described how sharing resources and staff allowed organizations to better leverage their own resources and human capital and prevented duplication of efforts to address the overdose crisis in the community.

## Discussion

In this study, we explored how health systems and CBOs develop and sustain partnerships to transition patients with SUD in hospitals to ongoing treatment. Using qualitative interviews with healthcare professionals and staff working in these settings, we found seven common themes that underlie the development of transition partnerships. These themes span from initial contact, maintaining the relationship and navigating disagreements, and factors important to sustaining partnerships. In establishing initial contact, interviewees emphasized the importance of active outreach, responding to community needs, leveraging reputation and networks, and having an external contact facilitate the introduction. To navigate the challenges that arise over the course of a partnership, interviewees discussed strategies such as maintaining a focus on daily operations rather than philosophical differences, using data to advocate for specific types of partnerships, as well as the benefits of a mutually beneficial and reciprocal relationship. Finally, in solidifying a partnership, participants described the utility of sharing data systems and employees between organizations.

Previous research on clinical partnerships endorses three key factors to guide partner selection: complementarity, commitment, and trust [27, 36–38]. Complementarity refers to the synergy of skills and resources between organizations, emphasizing collaboration over competition. Commitment encompasses tangible contributions from partners, such as investing time and resources, for the long-term success of the partnership. Trust refers to the ability of partners to overcome initial uncertainty and threats of opportunism [27]. The themes underlying transition partnership development that emerged from our study align well with these criteria for partnership selection. For example, in forging reciprocal relationships and responding to shared community needs, partners find skills and services that benefit each other and the community as a whole, which contributes to complementarity and reduces competition between partner organizations. Furthermore, focusing on operations, including overcoming disagreements surrounding patient care and sharing resources and infrastructure, represents tangible commitments from partners towards the relationship and demonstrates a willingness to make concessions to enable the relationship’s success. Finally, by engaging in active outreach, including traveling to meet potential partners in person, having an external facilitator guide initial introductions, and relying on positive reputations and mutual connections in a community, partners can create trust and overcome skepticism from partners. Relatedly, our results indicate that having data that demonstrates successful metrics and a history of partnership with other organizations can be used to build trust between organizations, as well as advocate for the development of new partnerships to fill specific needs.

A significant obstacle to establishing partnerships, specifically referral partnerships, amongst healthcare organizations treating SUD patients lies in divergent philosophies surrounding SUD. For example, previous research has found that healthcare organizations with a low-barrier or harm reduction approach to engaging with SUD patients may be unwilling to refer patients to care settings that lack such a philosophy [39, 40]. Although some strategies discussed by participants in this study, such as implementing shared data systems or hiring staff dedicated to forging clinical partnerships, may be difficult to implement, particularly in low-resource settings, strategies such as identifying ways to help each other, relying on previous relationships with partners, and focusing on daily activities could help organizations foster a culture of trust and overcome philosophical differences that may inhibit the development of successful partnerships. Indeed, several strategies discussed by interviewees in our study have been echoed in prior research describing the implementation of TOPs, including sharing staff between CBOs and hospitals [41–43] and maintaining open lines of communication between staff at both organizations [44]. 

Our study has both strengths and limitations that have implications for future work. Strengths include interviewing more than two dozen participants across four diverse safety net hospital organizations, which enabled us to achieve theoretical saturation. In addition, we included CBO participants which provided insight on the transition process from hospital to community-based treatment settings. Study limitations include our qualitative research design, which provides a depth of information on partnerships within study sites but limits generalizability. We selected four diverse safety net hospital organizations in terms of size, regional location, and development of transitional opioid programs. Still, future studies are necessary to determine if partnership experiences are similar at different types of hospital organizations. Finally, the CBOs interviewed in this study were referred to the study team by their health system/hospital partners and, as such, may be subject to selection bias. The study team also faced challenges establishing contact with CBO staff, and in particular was not able to establish contact with CBOs that had partnerships with one of the health systems in our study. As such, the perspective of CBO staff is underrepresented amongst participants, which skews our findings to the perspective of the health system respondents.

Our study builds on previous literature on developing effective health care-community partnerships by demonstrating how the establishment of such partnerships is accomplished for the successful transition of care for patients with OUD. Although there have been a number of studies describing the implementation of TOPs in hospital settings [18, 45–48] and evaluating the effectiveness of different types of TOPs [18, 46, 49–51], there has been a limited focus on how partnerships are developed between hospitals and CBOs. Prior studies have found that patients face difficulty transitioning from hospitals to community-based care [50]. Still, to our knowledge, the current study is the first to examine how partnerships to facilitate care transitions for SUD patients are established and maintained across multiple sites. This study is critical given that although hospitals are the frontlines of the overdose crisis [11, 13], as well as the healthcare entry point for many patients with SUD treatment [52], the majority of individuals who receive substance use treatment do so in community-based settings [3]. As such, it is critical to understand how patients can be transitioned between hospitals and outpatient or residential treatment in the community and, specifically, how partnerships that enable these linkages to occur are developed. The importance of these clinical partnerships is further underscored by the literature on ACOs, which finds that patients, including those with SUDs, have improved outcomes when receiving care in healthcare organizations that have formed partnerships with external entities [53, 54]. Findings from this study can assist hospitals and CBOs in forming partnerships that are specific to the needs of their SUD patient populations and the OUD treatment continuums. These findings can also support the implementation of TOPs within hospitals, as partnerships with external organizations that serve as transition endpoints are key stakeholders within the external implementation environment. For safety-net hospitals in particular, which operate on tight budgets [29, 30], developing partnerships and relationships with external organizations is a relatively low-cost activity that requires limited additional resources, as opposed to activities such as hiring care coordinators or developing new data systems. Beginning the TOP implementation process with a focus on partnerships may better enable TOP staff based in safety-net hospitals to advocate to hospital leadership for additional financial resources and supports to further develop TOPs.

Our findings also suggest strategies that may be useful to policymakers wishing to facilitate linkages for SUD patients in their communities, such as convening regional meetings for SUD treatment providers and organizations and investing in technologies and data systems that can better support these partnerships. Indeed, our results also highlight the important role of sustained public investments in supporting the adoption of these strategies–particularly for budget-constrained safety-net hospitals– as many of the programs included in our study received funding from public entities, including public health departments. These results are particularly salient given recent interest from federal and state governments in supporting care linkages for patients with SUD; for example, the Biden Administration recently announced plans to establish a set of hospital recommendations for overdose care and coordination and to create a model state law to further prioritize these strategies [55]. Future studies should evaluate how different partnership strategies translate to differences in partnership strengths and how this ultimately impacts patient outcomes, in both safety-net and non-safety-net settings. Given the importance of the patient voice, future research should also encompass the patient perspective and assess whether stronger partnerships between hospitals and CBOs improve patients’ experiences.

## Conclusions

This study identified seven core themes underlying the development of care transition partnerships for patients with OUD/SUD within four safety-net health systems and their CBO partners. These themes address different stages of relationship development, from initial formation to long-term sustainability, and demonstrate how partner organizations can establish the trust, reciprocity, and commitment necessary to support patients through the critical transition period. As health systems contend with the increasing severity of SUD and associated harms, the themes presented in this study may be useful as a guiding framework to establishing partnerships with other healthcare organizations that facilitate care coordination and transitions for patients with SUDs.

## Electronic supplementary material

Below is the link to the electronic supplementary material.

Supplementary Material 1

## Data Availability

No datasets were generated or analysed during the current study.
