# Supplementary material for: Forging hospital and community partnerships to enable care coordination for opioid use disorder
Source: Addict Sci Clin Pract. 2025 Apr 24;20:37. doi: 10.1186/s13722-025-00565-y (PMC12020205; doi:10.1186/s13722-025-00565-y)
Supplement: Supplementary file 1 — Supplementary Material 1 [file 13722_2025_565_MOESM1_ESM.docx]

**Appendix Table 1**. General Interview Guide

| **Interview Questions** | **Probes** |
| --- | --- |
| ***1. General background (be brief)***  1.1 For us to get to know you better, could you briefly describe your current role(s) at your organization? |  |
| ***2. Opioid use in the community (be brief)***  2.1 Could you briefly describe the current situation of opioid use in the community that your organization serves? |  |
| 2.2 What do you think is your organization’s approach to this current situation of opioid use that you have just described? | 2.2.a: Harm reduction orientation?  2.2.b: Grant-dependent? |
| ***3. Transitional Opioid Programs (for organizations that have TOPs)***  3.1 Some hospitals are involved in what we call Transitional Opioid Programs, which refers any effort to ensure that patients continue to receive services beyond treatment in the hospital. Can you briefly describe the TOPs or similar efforts in your organization? | 3.1.a: History or motivation behind TOPs (e.g., funding source, project origins)  3.1.b: General structure  3.1.c: Workflow (e.g., process – “warm hand-offs”, technology) and occupations/roles (e.g., champions) |
| 3.2. The referral network or relationships that your organization has with partners in the community is an important element of TOPs. Can you describe what those relationships are like? |  |
| 3.3 Can you share what makes these community partnerships work well, or not? | 3.3.a: “Bridge clinics”  3.3.b: History or motivation of partnerships  3.3.c: Short-term vs. long-term engagement  3.3.d: Ways to maintain partnership |
| ***4. Facilitators***  4.1 We are also interested to find out what makes TOP possible or successful based on your experience. Can you share what you think are some of the facilitators at the organizational level? What makes TOPs work, or work well? | 4.1.a: History or experience in implementing similar programs  4.1.b: ED-based care  4.1.c: Tracking/reporting of patient data |
| 4.2 Can you talk about the leadership, teams, or people that make TOPs possible or successful? | 4.2.a: Interprofessional teams  4.2.b: Having an Addiction Specialist or champion  4.2.c: Peers embedded in hospital  4.2.d: Professional education on OUD/SUD |
| 4.3 Can you share what you think are some of the facilitators at the broader environment or policy level? | 4.3.a: Resources/funding  4.3.b: County characteristics  4.3.c: Health care market characterstics  4.3.d: State and federal policy |
| 4.4. Do you think that some of these facilitators that you have shared can be replicated or should be considered by other organizations? If so, how? |  |
| ***5. Barriers***  5.1 We are interested to find out what hinders or challenges TOPs based on your experience. Can you share what you think are some of the barriers at the organizational level? What makes TOPs not work, or not work as well as expected? | 5.1.a: Lack of resources  5.1.b: Workflow challenges |
| 5.2 What about leadership, teams, or people that are involved? Is there something about the management of TOPs that is leading them to not work as expected?  As a reminder, this interview is confidential. |  |
| 5.3 Can you share what you think are some of the barriers at the broader environment or policy level? | 5.3.a: County characteristics  5.3.b: Health care market characterstics  5.3.c: State and federal policy (e.g., provider prescribing caps, provider licensing requirements)  5.3.d: Stigma  5.3.e: Abstinence requirement prior to Naltrexone |
| 5.4. Do you think that some of these barriers that you have shared can be avoided or should be considered by other organizations? If so, how? |  |
| ***6. Closing***  6.1 I will be asking you for contacts at the community partners that we will be able to speak to, and we will conclude today’s interview. Before I do that, is there anything else you would like to add? |  |
